# How community ART delivery may improve HIV treatment outcomes: Qualitative inquiry into mechanisms of effect in a randomized trial of community‐based ART initiation, monitoring and re‐supply (DO ART) in South Africa and Uganda

**DOI:** 10.1002/jia2.25821

**Published:** 2021-10-08

**Authors:** Hannah N. Gilbert, Monique A. Wyatt, Emily E. Pisarski, Stephen Asiimwe, Heidi van Rooyen, Janet Seeley, Maryam Shahmanesh, Bosco Turyamureeba, Alastair van Heerden, Oluwafemi Adeagbo, Connie L. Celum, Ruanne V. Barnabas, Norma C. Ware, for the Delivery Optimization of Antiretroviral Therapy (DO ART) Study Team

**Affiliations:** ^1^ Department of Global Health and Social Medicine Harvard Medical School Boston Massachusetts USA; ^2^ Harvard Global Cambridge Massachusetts USA; ^3^ Mbarara University of Science and Technology Mbarara Uganda; ^4^ Center for Global Health Massachusetts General Hospital Boston Massachusetts USA; ^5^ Integrated Community‐based Initiatives Kabwohe Uganda; ^6^ Human Sciences Research Council KwaZulu‐Natal South Africa; ^7^ SAMRC/Wits Developmental Pathways for Health Research Unit Department of Paediatrics Faculty of Health Sciences University of the Witwatersrand Johannesburg South Africa; ^8^ London School of Hygiene and Tropical Medicine London UK; ^9^ Africa Health Research Institute KwaZulu‐Natal South Africa; ^10^ Institute for Global Health University College London London UK; ^11^ Departments of Global Health and Medicine University of Washington Seattle Washington USA; ^12^ Department of Medicine Brigham and Women's Hospital Boston Massachusetts USA

**Keywords:** delivery of healthcare, antiretroviral therapy, quality of healthcare, Uganda, South Africa, HIV

## Abstract

**Introduction:**

UNAIDS fast track targets for ending the AIDS epidemic by 2030 call for viral suppression in 95% of people using antiretroviral therapy (ART) to treat HIV infection. Difficulties in linking to care following a positive HIV test have impeded progress towards meeting treatment targets. Community‐based HIV services may reduce linkage barriers and have been associated with high retention and favourable clinical outcomes. We use qualitative data from The Delivery Optimization of Antiretroviral Therapy (DO ART) Study, a three‐arm randomized trial of community ART initiation, monitoring and re‐supply conducted in western Uganda and KwaZulu‐Natal South Africa, to identify mechanisms through which community ART delivery may improve treatment outcomes, defined as viral suppression in people living with HIV (PLHIV).

**Methods:**

We conducted open‐ended interviews with a purposeful sample of 150 DO ART participants across study arms and study sites, from October 2016 to November 2019. Interviews covered experiences of: (1) HIV testing; (2) initiating and refilling ART; and (3) participating in the DO ART Study. A combined inductive content analytic and thematic approach was used to characterize mechanisms through which community delivery of ART may have contributed to viral suppression in the DO ART trial.

**Results:**

The analysis yielded four potential mechanisms drawn from qualitative data representing the perspectives and priorities of DO ART participants. Empowering participants to schedule, re‐schedule and select the locations of community‐based visits via easy phone contact with clinical staff is characterized as *flexibility*. *Integration* refers to combining the components of clinic‐based visits into single interaction with a healthcare provider. Providers” willingness to talk at length with participants during visits, addressing non‐HIV as well as HIV‐related concerns, is termed “*a slower pace*”. Finally, *increased efficiency* denotes the time savings and increased income‐generating opportunities for participants brought about by delivering services in the community.

**Conclusions:**

Understanding the mechanisms through which HIV service delivery innovations produce an effect is key to transferability and potential scale‐up. The perspectives and priorities of PLHIV can indicate actionable changes for HIV care programs that may increase engagement in care and improve treatment outcomes.

## 1 INTRODUCTION

UNAIDS fast track targets for ending the AIDS epidemic by 2030 call for viral suppression in 95% of people using antiretroviral therapy (ART) to treat HIV infection [1]. Recent UNAIDS estimates indicate that in eastern and southern Africa, 65% of people living with HIV (PLHIV) and prescribed ART have reached this milestone (64% in S. Africa and 75% in Uganda) [2]. A persistent gender gap pervades these data, as men are consistently less likely to attain viral suppression than women [2].

Difficulties in linking to care following a positive HIV test have impeded progress towards meeting treatment targets [3, 4]. Years of research has clarified the reasons for these difficulties. Long travel distances, the expense of transport, long waits to be seen by care providers and lost income due to time away from work remain major barriers to accessing care in HIV clinics [5, 6, 7, 8, 9], despite increasing the availability of ART. These barriers reduce ART initiation and adherence, and inhibit viral suppression.

Differentiated service delivery [10, 11, 12] may reduce these linkage barriers by moving care out of HIV clinics and into the community. To date, community‐based HIV care models have focused mostly on providing ART refills for individuals who are stable on ART and have suppressed viral loads. These include: (1) making medications available at fixed locations outside clinics, termed “community drug distribution points” [13]; (2) “adherence clubs”, which bring individuals together in communities for adherence support and refill pick‐up [14]; (3) decentralizing services to offer refills at primary care clinics closer to ART users” residences [15]; and (4) delivering ART refills at home [16]. Community‐based HIV services have been received favourably [17, 18], and are associated with high retention and favourable clinical outcomes [19, 20, 21, 22, 23, 24, 25]. We used qualitative methods to evaluate potential mechanisms contributing to increased viral suppression among people who received either community‐based or clinic‐based HIV care in the context of a randomized controlled trial.

## 2 METHODS

### 2.1 The Delivery Optimization for Antiretroviral Therapy Study

The Delivery Optimization for Antiretroviral Therapy (DO ART) Study offered ART initiation, monitoring and refills in communities to clinically stable adults living with HIV and not taking ART at the time of enrolment or within the previous 3 months. One year after study activation, detectable viral load was added as an inclusion criterion due to a higher than expected proportion of participants with suppressed viral load presenting at baseline. The DO ART Study randomized 1531 individuals to evaluate the relative effectiveness of: (1) community‐based ART initiation, monitoring and re‐supply via mobile vans; (2) clinic‐based ART initiation with community‐based monitoring and re‐supply (“hybrid” services); and (3) clinic‐based ART (standard of care). We hypothesized that community‐based ART would overcome logistical barriers to care, simplify monitoring and re‐supply, and increase viral suppression, particularly among men. DO ART was carried out at two peri‐urban sites in KwaZulu‐Natal, South Africa, and one site in rural Sheema District, in southwest Uganda.

Trained nurses and lay counsellors carried out DO ART Study activities. DO ART counsellors and nurses received training in “Nurse Initiation and Management of ART”, a task‐shifting approach to HIV treatment and care provision [26]. They also received specific training in the DO ART Study protocol, and clinical monitoring and supervision as they carried out the study. Mobile phones and airtime were provided to staff by the DO ART Study to facilitate communication with participants.

HIV testing and same‐day ART initiation were offered to participants randomized to the community‐based ART initiation, monitoring and refill study arm. Supplies of ART were dispensed for 1 month, 2 months, then every 3 months thereafter. Community‐based ART initiation and refill activities took place in mobile vans parked in designated locations at specified times in the community, and in other community venues (e.g. homes, dispensaries, community centres, athletic fields and workplaces). Participants randomized to hybrid services transitioned to community‐based follow‐up after clinic‐based ART initiation. Participants randomized to clinic‐based ART were referred to local clinics for treatment following HIV testing. The primary outcome for the DO ART Study was the proportion of participants with viral suppression at follow‐up month 12.

DO ART Study results revealed community‐based ART initiation and follow‐up increased viral suppression compared to clinic‐based ART services (SOC) (74% vs. 63%, RR = 1.18, 95% CI: 1.07–1.29); hybrid services were non‐inferior to clinic‐based care (68% vs. 63%, RR = 1.08, 95% CI: 0.98–1.19, *p* = 0.005 for non‐inferiority, RR>0.95). Both community ART initiation and follow‐up and hybrid services significantly increased viral suppression among men (community‐based ART and follow‐up: 73%, RR = 1.34, 95% CI: 1.16–1.55; hybrid services: 66%, RR = 1.19, 95% CI: 1.02–1.40) compared to clinic‐based care (54%) [27]. Annual per‐person cost of community‐based ART delivery was estimated at US$217 in Uganda and US$308–$312 in South Africa in the first year, and $187 in Uganda and $244–$246 in South Africa in subsequent years [27]. Community‐based ART was determined to be highly cost‐effective, at a cost of US$230 per DALY averted, compared to standard clinic‐based care [28]. Eighty‐four percent (84%) of participants in the DO ART randomized trial owned mobile phones [29].

The DO ART Study included a qualitative component charged with characterizing the intervention's mechanisms of effect [30, 31]. Mechanisms of effect may be understood as processes through which an intervention produces a given outcome, that is “how and why” an intervention works (or does not work). This paper draws upon qualitative data representing the perspectives and priorities of DO ART Study participants, to propose an explanation of how and why viral suppression may be increased through community delivery of ART.

### 2.2 Qualitative Methodology

#### 2.2.1 Sampling and data collection

We used purposeful sampling to identify participants in the qualitative component. Men and women representing all three study arms in all three DO ART Study sites were sampled, in approximately equal numbers. We oversampled participants in intervention (community ART initiation, monitoring and re‐supply; hybrid services) compared to control (clinic‐based care) arms. Qualitative participants were also selected to systematically represent 3, 6, 9 and 12 months of follow‐up at the time of their participation. More participants were sampled at later follow‐up points, to increase the overall intervention experience represented in the qualitative sample. Individual, in‐depth interviews were carried out with DO ART qualitative component participants by trained local research assistants (RAs). Interviews elicited information on experiences of: (1) HIV testing; (2) initiating and refilling ART; and (3) participating in the DO ART Study. Interviews were carried out in local languages, in convenient, private locations and lasted 45–60 min on average. At the conclusion of each interview, participants were reimbursed in local currencies at rates commensurate with local standards and practices (amounts ranging from ∼USD $2.00 to $6.00). Interviews were audio‐recorded, with permission. Recordings were transcribed directly into English by the RA who conducted the interview. Interview transcripts were regularly reviewed to monitor data quality. RAs participated in weekly calls and emails for feedback on interviewing and transcription technique. Qualitative data collection began in October 2016 and ended in November 2019.

#### 2.2.2 Data analysis

A combined content analytic and thematic approach [32, 33] was used to analyse the qualitative data, with the goal of characterizing mechanisms through which community‐based delivery of ART may have promoted viral suppression in the DO ART Study. From DO ART participants” descriptions of their experiences receiving ART in their communities, we inductively developed concepts characterizing potential mechanisms of effect. These mechanisms are described in Results.

Transcripts were reviewed by authors MW and EP to develop a coding scheme. We used open coding to identify and label relevant content from interview transcripts. Labels were operationally defined, reviewed, refined and assembled into a codebook. To ensure coding consistency, two coders (author EP and an RA) each coded 25% (*N* = 37) of the transcripts, and identified and resolved discrepancies through discussion. The remainder of the dataset was singly coded using Atlas.ti qualitative management software (https://atlasti.com). The coding process reduced the data to yield an analytic dataset focused on experiences of receiving community ART.

In addition to coding, individual participants” experiences of receiving ART in the community were summarized and entered into a matrix. The matrix both preserved the coherence of individual experiences and helped to identify patterns that cut across the data, for a thematic analytic approach. The thematic analysis was carried out by lead author HG, and resulting themes were reviewed and agreed upon by NW, EP and MW.

Thematic concepts identified through the matrix were elaborated through coded data. Coded data corresponding to these concepts were retrieved and used by HG to expand, revise and add detail to the thematic concepts. The revised concepts were then labelled, described and illustrated to form descriptive categories, which were reviewed and finalized by HG and NW. By employing this combined thematic and content analytic approach, we arrived at a set of categories representing a variety of potential mechanisms based on the perspectives and priorities of DO ART Study participants.

### 2.3 Ethical approval

Approval to carry out the qualitative research was obtained from the Committee on Human Studies, Harvard Medical School, Boston, MA; the University of Washington Institutional Review Board, Seattle, WA; the Human Sciences Research Council Research Ethics Committee, Pretoria, South Africa; Mbarara University of Science and Technology Research Ethics Committee, Mbarara, Uganda; and the Uganda National Council for Science and Technology, Kampala, Uganda. Participants provided consent for qualitative interviews as part of the DO ART Study consent process. Consent was re‐confirmed verbally as part of recruitment for the qualitative interviews.

## 3 RESULTS

### 3.1 Qualitative participants

One‐hundred‐fifty (*N* = 150) DO ART Study participants took part in the qualitative component. Fifty‐eight (*N* = 58) interviewees were in the community‐based ART initiation, monitoring and re‐supply arm; 62 were in the hybrid services arm; and 30 were in the clinic‐based care arm. Fifty‐one interviewees were from Uganda; and 99 were from South Africa, where there were two DO ART Study sites. Eleven were sampled and interviewed at month 3, 28 at month 6, 36 at month 9 and 75 at month 12.

Men and women are nearly equally represented in the qualitative sample, reflecting our purposeful sampling strategy. The majority of participants were between the ages of 30 and 49, and had some secondary education. About two‐thirds were in partnered relationships. More than half were engaged in income‐generating activity. HIV testing at DO ART enrolment yielded the first HIV‐positive test result for about three‐quarters of participants. Approximately 80% had achieved viral suppression at DO ART Study exit. There were no major differences in personal characteristics across qualitative sub‐groups defined by intervention arm (Table 1).

**Table 1:** Personal characteristics of qualitative participants (*N* = 150)

	Total sample (*N* = 150)	Community ART initiation, monitoring and re‐supply arm (*N* = 58)	“Hybrid” services arm (*N* = 62)	Clinic‐based care arm (*N* = 30)
	*N*/%	*N*/%	*N*/%	*N*/%
Sex				
Male	76 (51%)	31 (53%)	30 (48%)	15 (50%)
Female	74 (49%)	27 (47%)	32 (52%)	15 (50%)
Age				
18–29	58 (39%)	21 (36%)	27 (44%)	10 (33%)
30–49	84 (56%)	34 (59%)	32 (52%)	18 (60%)
>50	8 (5%)	3 (5%)	3 (5%)	2 (7%)
Education				
No school/unknown	6 (4%)	1 (2%)	5 (8%)	1 (3%)
Primary	51 (34%)	21 (36%)	19 (31%)	11 (37%)
Secondary	88 (59%)	34 (59%)	36 (58%)	18 (60%)
Tertiary	4 (3%)	2 (3%)	2 (3%)	0 (0%)
Relationship status				
In a partnered relationship, married	103 (69%)	42 (72%)	40 (65%)	21 (70%)
Income generation				
Some income‐generating activity	87 (58%)	35 (60%)	34 (55%)	18 (60%)
HIV testing				
Tested for HIV before DO ART	89 (59%)	41 (71%)	32 (52%)	16 (53%)
First HIV‐positive test at enrolment	114 (76%)	43 (74%)	49 (79%)	22 (73%)
Viral suppression^a^				
At DO ART study exit	98/124 (79%)	38/47(81%)	42/54 (78%)	18/23 (78%)

^a^ Participants purposefully sampled as being virally suppressed at baseline and those for whom data on viral suppression could not be confirmed at exit are excluded here.

### 3.2 Qualitative results

Our thematic, content analytic approach privileging the perspectives of study participants yielded core concepts suggesting mechanisms through which community‐based ART initiation, monitoring and re‐supply may have positively impacted viral suppression in the DO ART Study. The concepts are: (1) flexibility; (2) integration; (3) “a slower pace”; and (4) increased efficiency. Data from virally suppressed and non‐virally suppressed participants were included in the analysis.

#### 3.2.1 Flexibility

Community‐based ART delivery was distinguished by its flexible approach to visits. Staff shared phone contact information with participants and routinely called with reminders of upcoming appointments and to fix meeting times and places. Because they had phone numbers, participants could also easily contact staff to re‐schedule meeting times when the need arose. Participants were also welcome to contact staff for other reasons, and they did not hesitate to be in touch when they were running low on ART, had questions or needed general health advice. Easy phone contact between participants and staff cut down on missed meetings and promoted continuity, reducing the risk of adherence lapses resulting from running out of pills.

The prerogative of contacting staff was greatly appreciated by participants, who interpreted it as a sign of caring on the part of the healthcare system. Staff willingness to meet participants wherever they were in the community was also very highly valued, as it increased confidence that medications would be reliably obtained.

Inviting participants to choose locations of follow‐up visits also reflected flexibility. When contacting participants to with reminders, staff would also encourage them to suggest a meeting place. Thus, participants could draw on their knowledge of communities to select locations where interactions were unlikely to be observed. This preserved privacy and reduced the risk of unwanted disclosure and resulting stigma.

The mechanism of flexibility is evidenced in the following quotes:

“Getting my drugs from the community was easy for me because it would take a short time and there was flexibility. If I were to go for my drugs like at 10:00 am and I get another program [something comes up], we would agree and go at around 2:00 pm and I would still get my drugs without any challenge”. Man, Age 25, Uganda, Hybrid arm

“If I had started [ART] from the clinic my life was going to be difficult. Perhaps I would have defaulted due to the challenge of walking. … It was a better plan that they come to my house, because sometimes when they came I would tell them that today I am not feeling well. And then the staff would end up coming inside the house. Normally when they come I go inside the mobile van. And they will then do what they are meant to do and they will leave when they have finished”. Woman, Age 44, South Africa, Community ART initiation monitoring & re‐supply arm

“We met at the football pitch below this village. She [DO ART staff] called me and told me that she was coming and we agreed that she finds me at the football pitch. We met there. It's me who decided that we meet at that place. I chose that place because nobody would see me and it is spacious enough, with parking space in case she was to come with the van”. Woman, Age 23, Uganda, Hybrid arm

#### 3.2.2 Integration

DO ART Study visits consisted of a single interaction between staff and participants. Unlike clinic‐based care, where every procedure typically has its own assigned space, administering health worker and associated queue, in the DO ART Study, all HIV treatment activities required for delivering ART were “integrated” into one encounter. Integrated visits were enthusiastically received by participants, who valued their greater efficiency and privacy. Efficiency and privacy gains were seen as resulting from elimination of the need for repeated queuing. Not having to wait in line for each procedure saved time and reduced the risk of being “outed” as someone living with HIV.

The DO ART Study's integrated approach made keeping appointments for HIV services easier and more appealing, compared to visiting the clinic. Appointments were thus more likely to be kept, and participants were less likely to run out of medication.

“[The van] is simple. It's just that everything you do, it's in one room. But at the clinic, you go for blood tests – there is a queue there. You go for the scale [for weighing], you are also gonna be in a queue. You see that? But in the van, you do everything in one room, and you get your treatment in one room”. Man, Age 33, South Africa, Community ART initiation, monitoring & re‐supply arm

“I would find him [RA] at the office at any time and get my drugs…This is contrary to getting drugs from the clinic because it involves many procedures, you have to go through several offices. Yet in the community refill, you see only one person and get your drugs in a short time and go back home early. With the clinic refill, you go to the clinic early in the morning like at around 8:00 am but spend there a lot of time and go back home at around 2:00 pm but with community refill, the research assistant asks you all the questions he wants to ask you and does all the procedures within like 30 minutes and gives you your drugs and you go back home early”. Man, Age 32, South Africa, Community ART initiation, monitoring & refill arm

#### 3.2.3 “A slower pace”

Participants experienced their community‐based visits with DO ART staff as relaxed – often in sharp contrast to clinic experiences. In DO ART visits, lay counsellors took the time to talk at length about participants” experiences and concerns. This unhurried atmosphere encouraged questions, and the airing of worries about physical problems and larger life challenges. Counsellors responded by offering tailored counselling, seeking outside advice and referrals, and/or accompanying participants to points of care.

“[I liked]…the warm welcome they gave us, and that they told us to feel free and open to ask any questions we like to ask and they will answer you correctly. If someone among the [staff] is not sure or couldn't explain well, she would ask someone who is able to explain properly and you just get out understanding. Do you know that being explained properly can heal your soul and when you take ARVs you just feel right?” Woman, Age 32, South Africa, Hybrid arm

“I always call him [counselor] in case I want to ask him anything and he explains to me and I feel satisfied…What I like about it is because he discusses with me so often. He is open and explains to me whatever I ask him even if it's not related to HIV… I asked him how I can go about my fracture and he advised me to go to [name of hospital] and see specialists that can help me”. Man, Age 28, Uganda, Community ART initiation, monitoring & re‐supply arm

Participants reported that the counsellors” cheerful attitudes brought a lightness and sense of hope to clinical encounters. Visits left them in a positive frame of mind, feeling emotionally fortified, free from stress and worry, and encouraged to succeed at taking ART.

“[When] I left from the van… I had oomph, because I had courage. I told my partner that it's like I am in the new phase, and I am going to fight this thing because of the strength I got from the caravan”. Man, Age 66, South Africa, Community ART initiation, monitoring, and re‐supply arm

#### 3.2.4 Increased efficiency

Participants found community ART increased HIV treatment efficiency by reducing the time needed to travel to, keep and travel back from clinical appointments. Reduced time eliminated conflicts between the demands of treatment persistence and income generation requirements. A broad spectrum of occupations was represented in this qualitative sample, which included farmers and other day labourers, formal employees and business owners. All expressed appreciation for the ready access to ART with shorter time investments that came with community ART.

For the lowest earners, losing time for income generation was a grave concern. Day labourers explained that a visit to clinic meant paying for transportation while also losing an entire day's paid work – a double financial burden. Receiving ART in the community, in contrast, eliminated transport costs and freed up time to earn a day's wages.

“I want to extend my thanks to those people who suggested that we should collect our drugs in our villages because it saves time and it does not involve transport costs. For us, we don't work in offices so we don't earn salary. Therefore when you go to clinic and you spend the whole day at clinic without doing any work, you can't eat”. Man, Age 32, Day laborer, Uganda, Hybrid arm

Participants working outside the day labour economy also spoke frequently about the financial strain of clinic‐based visits. Salaried employees complained that supervisors often did not accept clinic appointments as a justifiable reason for absence from work, and docked pay, creating considerable stress. Community ART delivery enabled employees to take a quick break from work to keep appointments, then quickly return to their jobs.

“I like the fact that they deliver [the medication] at home, because with the work that I do, sometimes my supervisor disrespects us. Sometimes … when she hears that you were not at work the previous day, and you explain to her that you were at the clinic to collect you pills, she just treats you as if you were absent and you don't get fully paid”. Woman, Age 30, South Africa, Community ART initiation, monitoring & re‐supply arm

“…when they refill you from home they make things easier. If you go to the clinic, definitely you won't be able to make it at work. It is unlike in other types of jobs, where if you did not go to work you [still] get paid, for my job if you do not go to work you miss a payment. … Even if you submit a letter that you were at the clinic, they do not work like that”. Man, Age 39, South Africa, Community ART initiation, monitoring & re‐supply arm

Self‐employed business owners echoed these sentiments. While not dependent upon supervisors to excuse absences, business owners felt uncomfortable shuttering their establishments for hours in order to keep clinic appointments. Being closed meant losing income; to be successful, businesses needed to remain open. Community ART delivery enabled business owners to quickly and conveniently pick up medications and return to work, eliminating the need for extended closures.

“Operating a salon is a job that you can't just close and go away. It needs a lot of time if you are to make some money. Closing it for the whole day that you are going to the clinic means that you will not make any money that day yet you have to spend. [Community‐based ART] was therefore my preference, because I knew I would start my drugs in my home and rush back to my job”. Man, Age 28, Uganda, Hybrid arm

## 4 DISCUSSION

This qualitative analysis sought to explain how the DO ART Study model of community ART delivery worked to increase viral suppression in Ugandan and South African adults living with HIV, compared to standard clinic‐based care. Drawing on participants” descriptions of community‐based services, the analysis identified four intervention mechanisms that may have contributed to increased viral suppression rates with community ART. Visit *flexibility* in the form of easy re‐scheduling of appointments appeared to reduce the risk of missed refills and consequent treatment lapses. *Integration* simplified the process of keeping appointments, promoting continuity in medication re‐supply and daily dosing. A *slower pace* of visits increased staff time for answering questions and offering encouragement, thereby helping sustain participants” determination to succeed at ART. *Increased efficiency* reduced time needed for visits, making it easier to combine treatment persistence with income generation. All of these mechanisms represent potential contributors to ART adherence and thus, to favourable rates of viral suppression among individuals randomized to receive ART in communities.

Characterizing the mechanisms through which an intervention works sheds light on its transferability to other settings. Almost by definition, bringing ART to communities, near where users live, eliminates or greatly reduces distance and associated transport costs as access barriers. This suggests a good fit for scale up in rural settings. More prominent than ease of access in the reports of these qualitative participants, however, were improvements to service quality that saved time and made receiving care simpler and more pleasant. These improvements facilitated medication re‐supply and subsequent adherence. Fewer clients per provider may be necessary to slow the pace of visits, requiring an increase in human resources and associated costs. Integrated visits and other structural changes, such as increasing the interval between refill appointments, could free up staff time and save on personnel costs. At this juncture, all of the mechanisms described here appear affordable and potentially scalable, suggesting that community ART may be transferable to a wide variety of HIV care settings.

In their comment on DO ART Study results, Nachega et al. [34] note the importance of attending to the “adjunctive practices” accompanying interventions that may impact external validity. Phone reminders of upcoming appointments, “facilitated re‐scheduling”, and follow‐up phone conversations are cited as examples of such adjunctive practices. The significance of adjunctive practices is underscored by our qualitative analysis, which points to the availability of easy phone contact as a core ingredient of the mechanism we term *flexibility*. Nachega et al. caution that scale‐up of community‐based ART without adjunctive practices included in prior effectiveness trials could dilute its real‐world impact. No appreciable obstacles to implementation of regular staff–participant phone communication as part of community ART delivery were evident in the qualitative data. Both staff and participants relied on phone calls as a simple and effective means of promoting continuity and supporting ART adherence.

DO ART Study results revealed that the positive impact of community delivery of ART on viral suppression was greater for men than women [27]. Qualitative data suggest that one reason for this may be the alleviation of conflict between the demands of income generation and keeping clinic‐based appointments – here termed *increased efficiency*. Across occupations, men endorsed the importance of relief from this conflict for their ability to remain in HIV care. Our data do not allow for quantification of sex differences; however, greater pressures on men to earn income for family support may translate to relatively greater increases in treatment persistence resulting in increased efficiency, and thus to higher rates of viral suppression.

Qualitative methods are not well‐suited to the examination of subgroup differences overall, since their aim tends to be synthetic – aimed at “grouping” rather than “splitting” – to characterize patterns in textual data. Major differences can occasionally be observed, however. Here, appreciation for integration and the slower pace of community‐based care appeared more prominent among South African participants. This makes sense in light of the greater population density and higher HIV prevalence (estimated at 36%) in the Kwazulu‐Natal study settings [35], which may translate to more crowded conditions at HIV clinics.

Some of the concepts presented here have been reported in other qualitative studies. Reduced time and expense of services, and improved quality and support for ART adherence are noted as benefits in a recent qualitative evaluation of community ART refill groups in Zimbabwe [36]. In a study of patient experiences of ART adherence clubs in Cape Town, South Africa, reduced time and increased flexibility of appointments were cited as advantages [18]. Our analysis is distinct in examining participant experiences for intervention mechanisms and explicitly drawing out implications for transferability and scale up.

The four mechanisms described here provide a close‐up look at how public health services may be adapted to meet the diverse needs of PLHIV in sub‐Saharan Africa. The mechanisms need not be treated as a package, but may be mixed and matched to fit particular service contexts. Some may also be compatible with clinic‐based as well as community‐based care. The DO ART Study model responds to the call for more personalized services tailoring care to individuals, so as to engage all in need in HIV care [37].

We acknowledge the limitations of this study. While the sample size is quite large for qualitative research, it does not include constituencies other than DO ART Study participants, for example staff who implemented the intervention. We do not claim the set of mechanisms we have identified to be exhaustive; others not accessible by investigating the perspectives of participants may also have contributed to DO ART Study outcomes. Similarly, the analysis does not discuss mechanisms which may have detracted from the effectiveness of the intervention, or which may help to explain differences in outcomes across intervention arms. Experiences of ART initiation in the community are not represented in this analysis; the mechanisms presented apply largely to monitoring and refill experiences.

## 5 CONCLUSIONS

This analysis identified four mechanisms through which community delivery of ART may have resulted in higher rates of viral suppression for adults living with HIV and not taking ART. All point to seemingly actionable changes in service organization that appear to reduce treatment lapses and increase medication adherence by improving quality. Understanding the mechanisms through which HIV service delivery innovations produce an effect is key to evaluating transferability and potential scale‐up.

## COMPETING INTERESTS

The authors declare that they have no competing interests.

## AUTHORS’ CONTRIBUTIONS

MW and NW designed the research. MW and EP designed the data collection instruments. SA, TB, HvR, AvH, JS and OA supervised collection of the data at Ugandan and South African DO ART Study sites. HG analyzed the data and drafted the Results. RB, CC and MS provided early feedback on the direction of the analysis reported in Results. NW drafted the remainder of the manuscript and made revisions. All authors read and approved the final manuscript.

## Data Availability

The qualitative data supporting the findings of this study have been deposited in Harvard Dataverse, a repository for research data (https://doi.org/10.7910/DVN/DQWJMF). Data are available to researchers who meet criteria for access.
